# Supplementary material for: Activation of the insulin receptor by an insulin mimetic peptide
Source: Nat Commun. 2022 Sep 23;13:5594. doi: 10.1038/s41467-022-33274-0 (PMC9508239; doi:10.1038/s41467-022-33274-0)
Supplement: Supplementary file 1 — Supplementary Information [file 41467_2022_33274_MOESM1_ESM.pdf]

Supplementary information

Activation of the insulin receptor by an insulin mimetic peptide

Junhee Park, Jie Li, John P. Mayer, Kerri A. Ball, Jiayi Wu, Catherine Hall, Domenico Accili, Michael H.B. Stowell, Xiao-chen Bai, Eunhee Choi

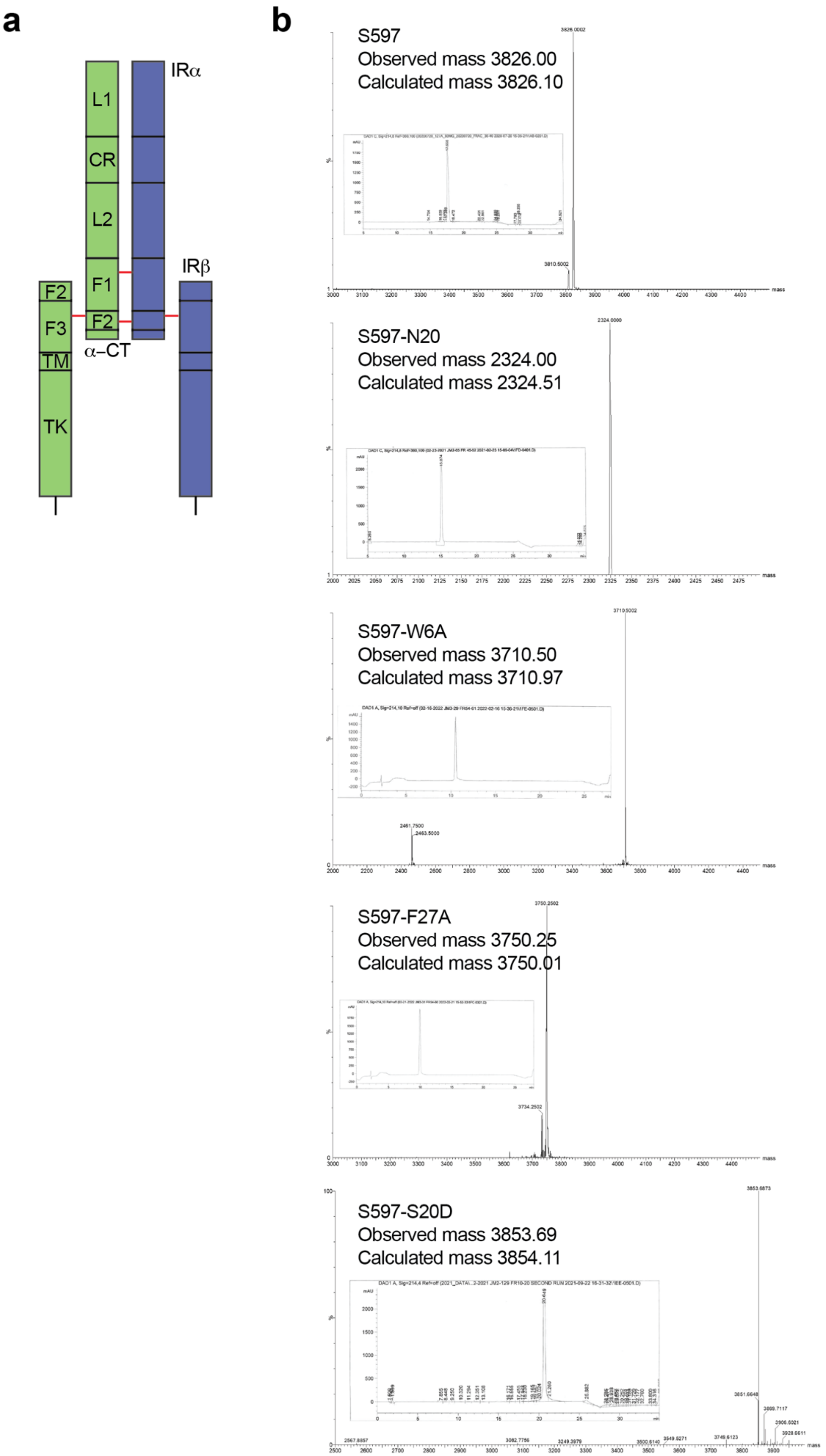
a
L1
CR
L2
F1
F2
IRα
IRβ
F2
F3
TM
TK
α–CT
b
S597
Observed mass 3826.00
Calculated mass 3826.10
S597-N20
Observed mass 2324.00
Calculated mass 2324.51
S597-W6A
Observed mass 3710.50
Calculated mass 3710.97
S597-F27A
Observed mass 3750.25
Calculated mass 3750.01
S597-S20D
Observed mass 3853.69
Calculated mass 3854.11

Supplementary Figure 1. Domains of insulin receptor (IR) and preparation of S597 peptides.

a. Domain structure of IR. L1 and L2, leucine rich domains 1 and 2; CR, cysteine rich domain, F1, F2, and F3, fibronectin III (FnIII) domains; TM, transmembrane domain; TK, tyrosine kinase domain; α-CT, C-terminal region of IRα. Red lines indicate disulfide bonds.

b. HPLC traces and MS1 spectra for S597 and S597 mutant peptides synthesized and utilized for both functional and structural studies.

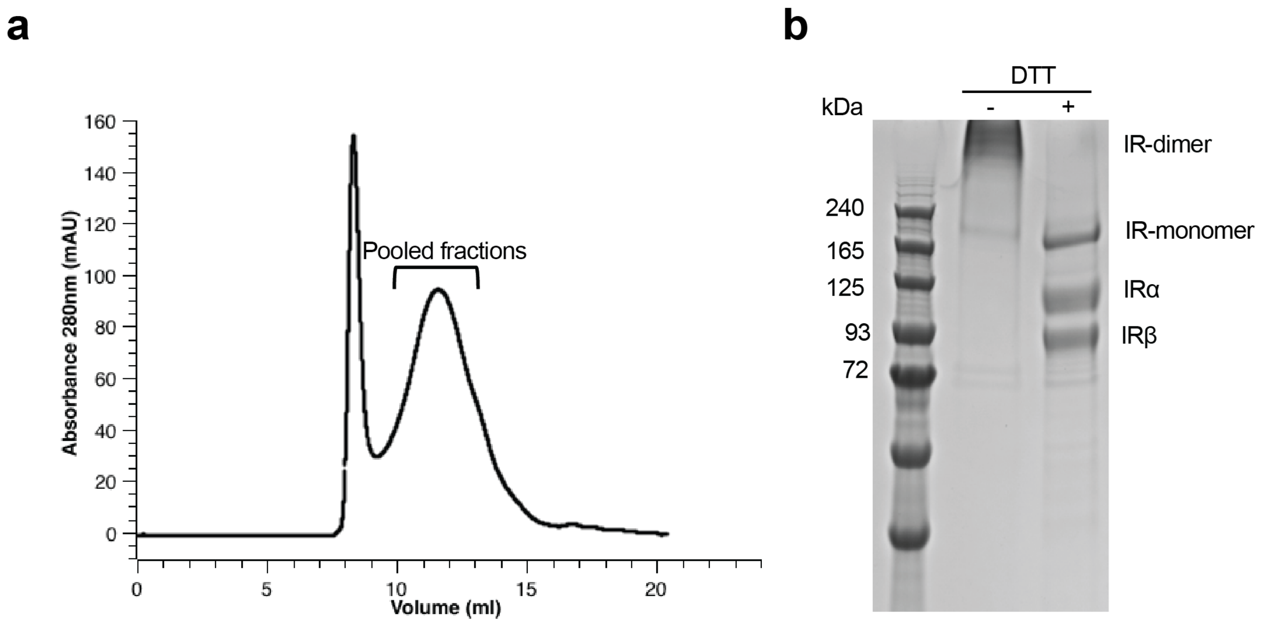

Supplementary Figure 2. Preparation of IR proteins.

a. A representative size-exclusion chromatography of IR.

b. The peak fractions were combined and visualized on SDS-PAGE by Coomassie staining, in the absence or presence of dithiothreitol (DTT). Experiment was repeated ten times.

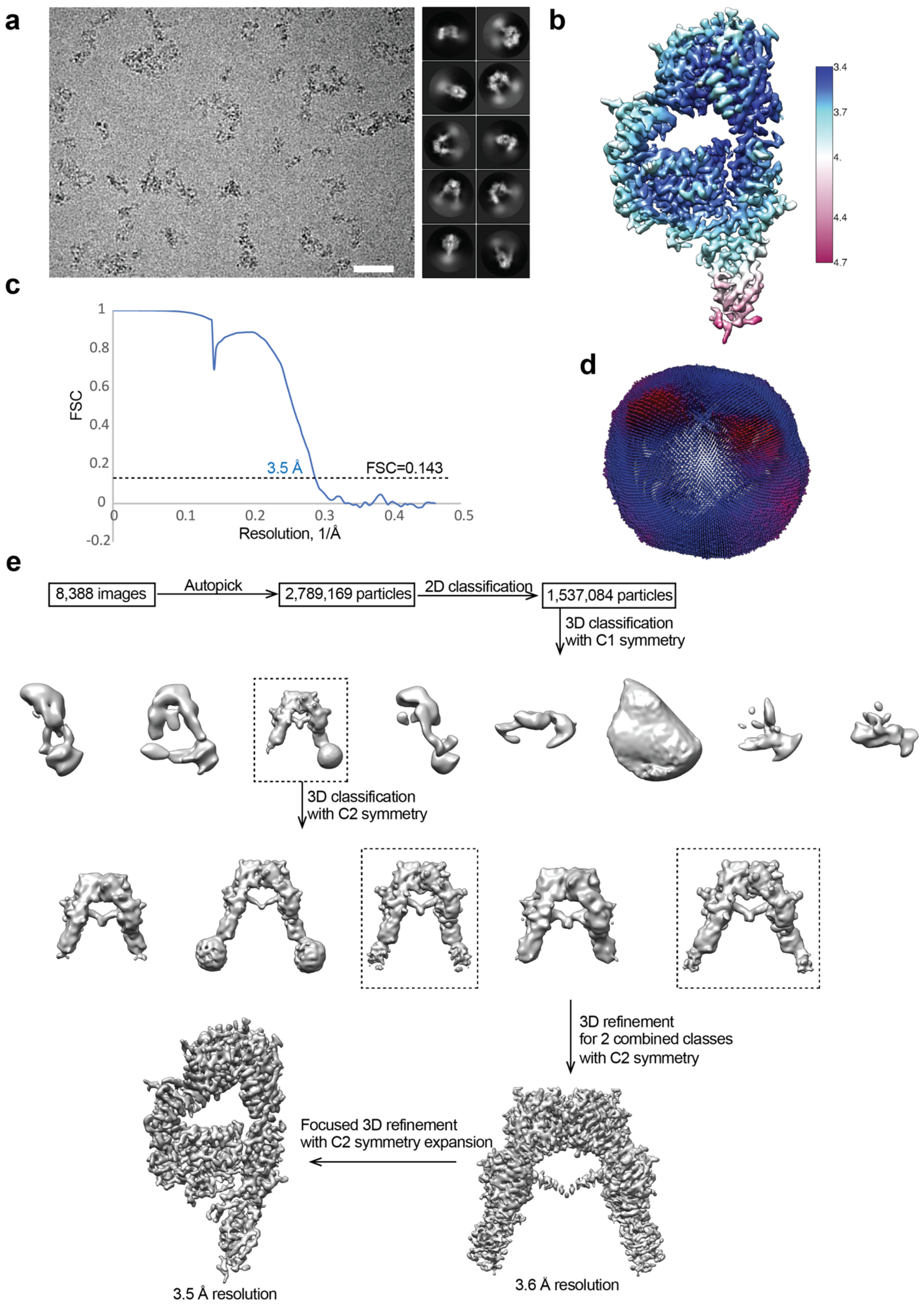

a
b
3.4
3.7
4.
4.4
4.7
c
1
0.8
0.6
0.4
0.2
0
-0.2
FSC
3.5 Å
FSC=0.143
0
0.1
0.2
0.3
0.4
0.5
Resolution, 1/Å
d
e
8,388 images
Autopick
2,789,169 particles
2D classification
1,537,084 particles
3D classification
with C1 symmetry
3D classification
with C2 symmetry
3D refinement
for 2 combined classes
with C2 symmetry
Focused 3D refinement
with C2 symmetry expansion
3.5 Å resolution
3.6 Å resolution

Supplementary Figure 3. Cryo-EM analysis of the IR/S597-N20 complex.

a. Representative electron micrograph and 2D class averages of the IR/S597-N20 complex. Scale bar: 300 Å. 8388 images were collected.

b. Unsharpened cryo-EM map colored by local resolution.

c. The gold-standard Fourier Shell Correlation (FSC) curve for the cryo-EM map shown in **Fig. 1**.

d. Angular distribution of particles used in the final 3D reconstructions.

e. Flowchart of cryo-EM data processing.

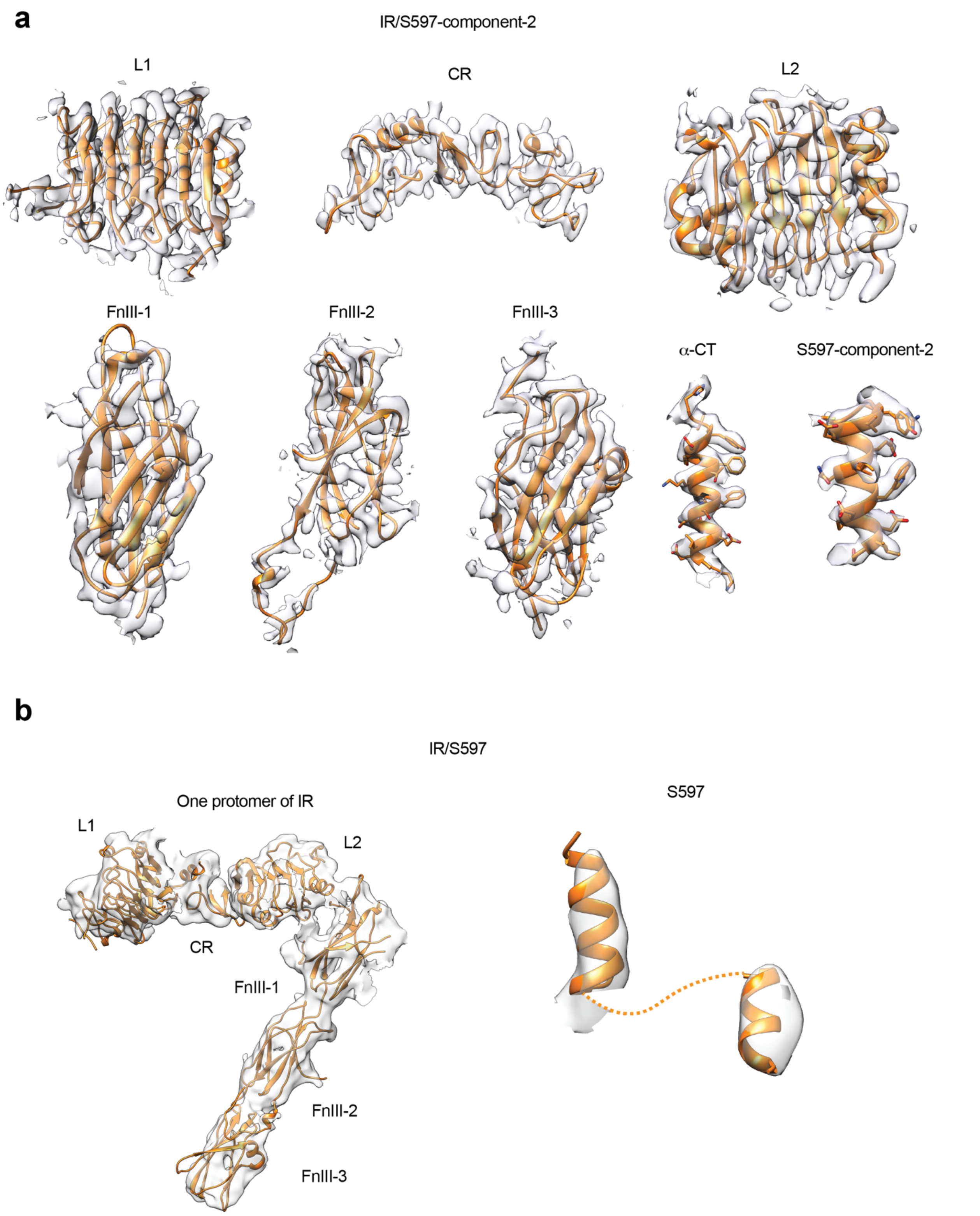

Supplementary Figure 4. Cryo-EM densities of the IR/S597-component-2 and IR/S597 complex.

a. Representative densities of the cryo-EM map of each domain of IR and S597-component-2.

b. Representative densities of the cryo-EM map of one protomer of IR and S597.

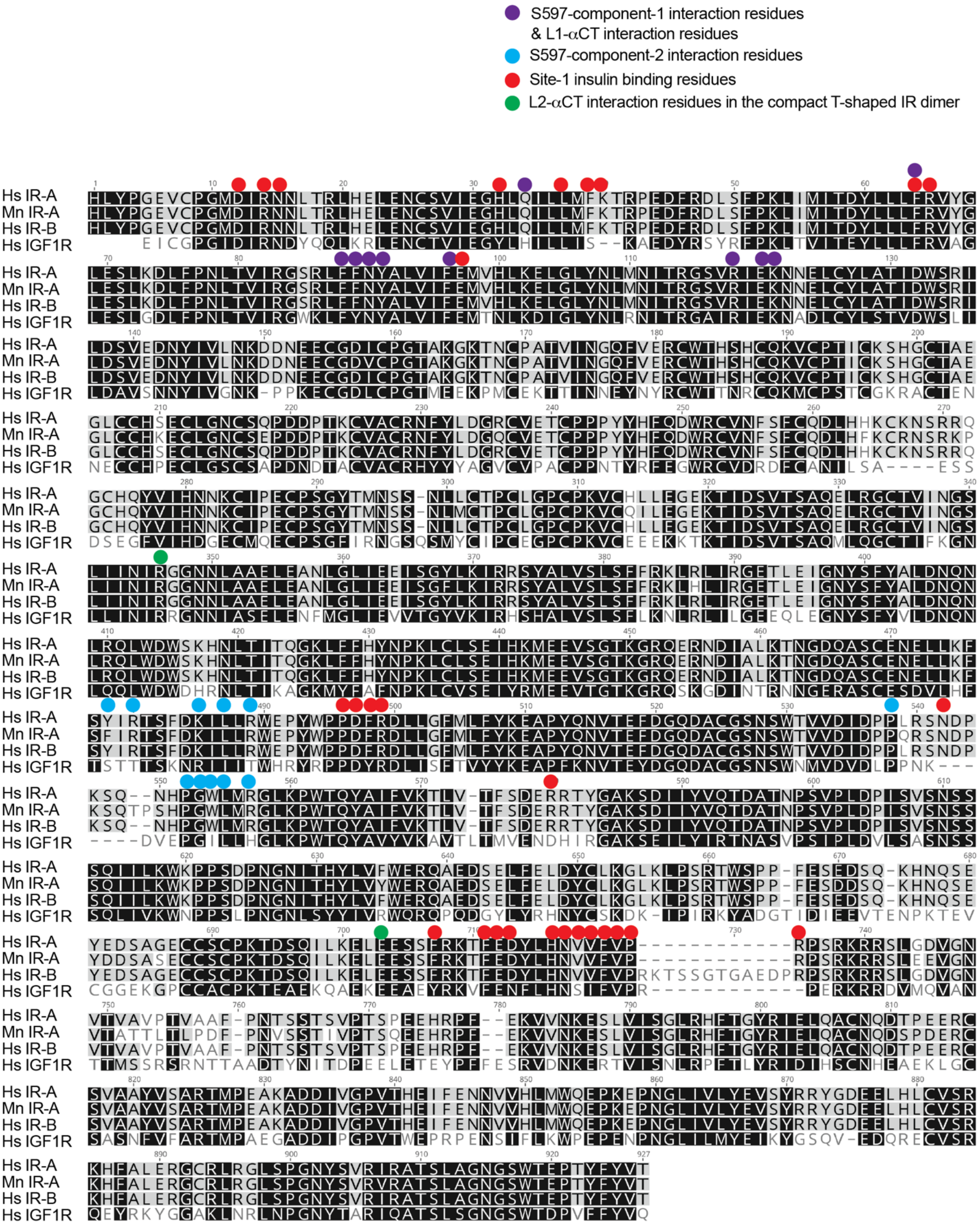

Supplementary Figure 5. Sequence alignment of IR proteins from human (Hs), mouse (Mn), and Hs IGF1R. IR-A and IR-B indicate A isoform (short) and B isoform (long) of the IR, respectively. Key residues are marked.

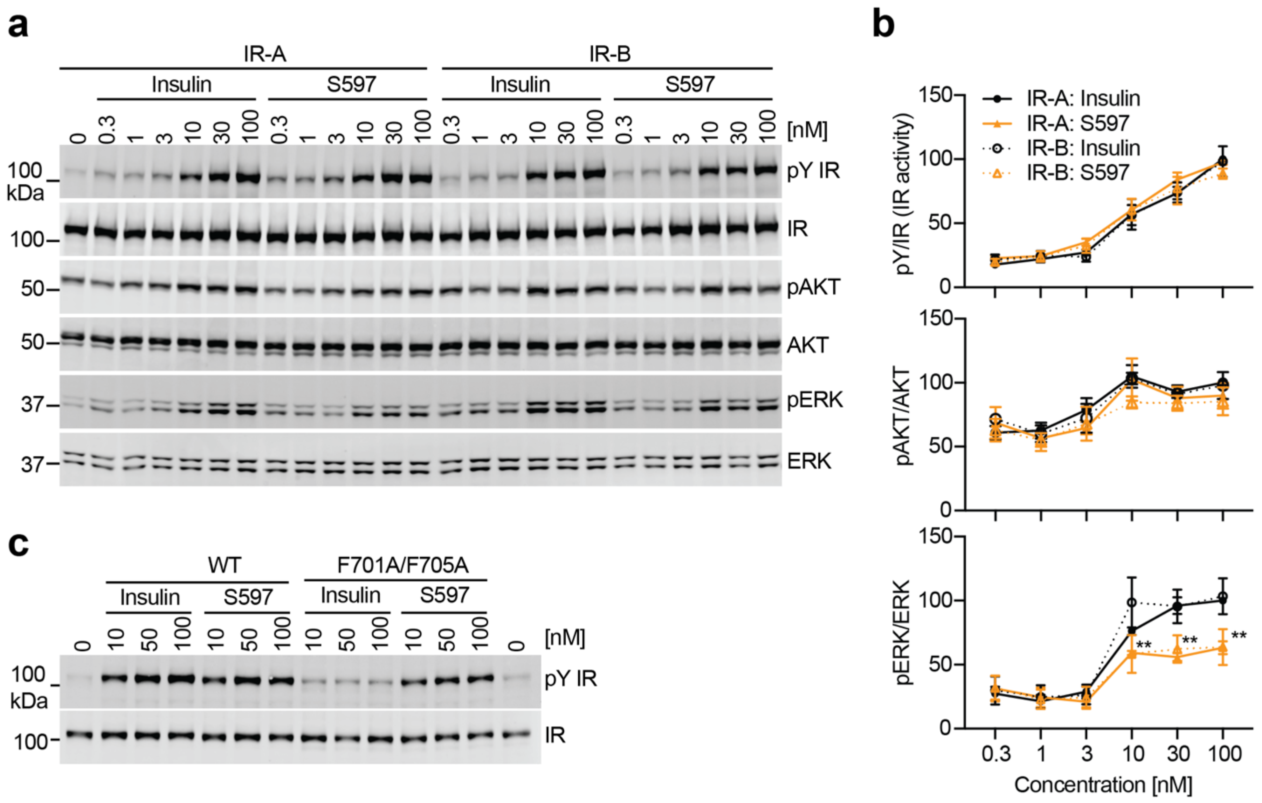

Supplementary Figure 6. S597-induced IR activation.

a. IR signaling by the indicated concentrations of insulin or S597 for 10 min in 293FT cells expressing IR-A or IR-B.

b. Quantification of the western blot data shown in (**a**). Levels of protein phosphorylation were normalized to total protein levels and shown as intensities relative to IR-A in 100 nM insulin-treated cells. Mean ± SEM. Each experiment was repeated three times. Significance calculated using two-tailed Student's *t-test*; IR-A between insulin and S597 in the indicated concentrations with the IR-A set as the control. **p<0.01. The exact p values are provided in the source data.

c. The α-CT motif of IR is not necessary for S597-dependent IR activation. Auto-phosphorylation of IR by the indicated concentrations of insulin or S597 for 10 min in 293FT cells expressing IR wild-type (WT) or F701A/F705A mutant. Quantification of the western blot data is shown in **Fig. 4d**. Source data are provided as a Source Data file.

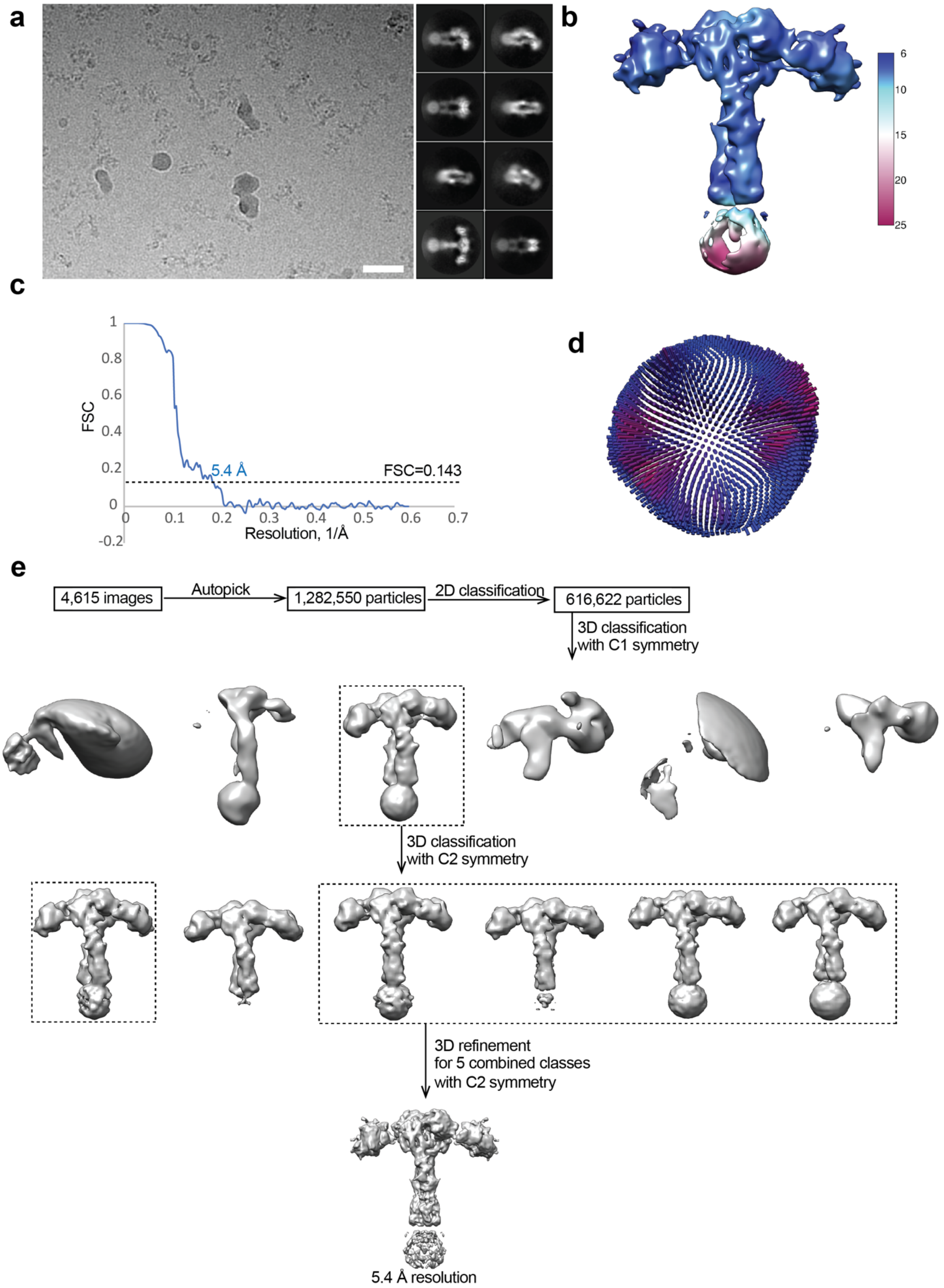
a
b
6
10
15
20
25
c
1
0.8
0.6
0.4
0.2
0
-0.2
FSC
5.4 Å
FSC=0.143
0
0.1
0.2
0.3
0.4
0.5
0.6
0.7
Resolution, 1/Å
d
e
4,615 images
Autopick
1,282,550 particles
2D classification
616,622 particles
3D classification
with C1 symmetry
3D classification
with C2 symmetry
3D refinement
for 5 combined classes
with C2 symmetry
5.4 Å resolution

Supplementary Figure 7. Cryo-EM analysis of the IR/S597 complex.

a. Representative electron micrograph and 2D class averages of the IR/S597 complex. Scale bar: 300 Å. 4615 images were collected.

b. Unsharpened cryo-EM map colored by local resolution.

c. The gold-standard Fourier Shell Correlation (FSC) curve for the cryo-EM map shown in **Fig. 3**.

d. Angular distribution of particles used in the final 3D reconstructions.

e. Flowchart of cryo-EM data processing.

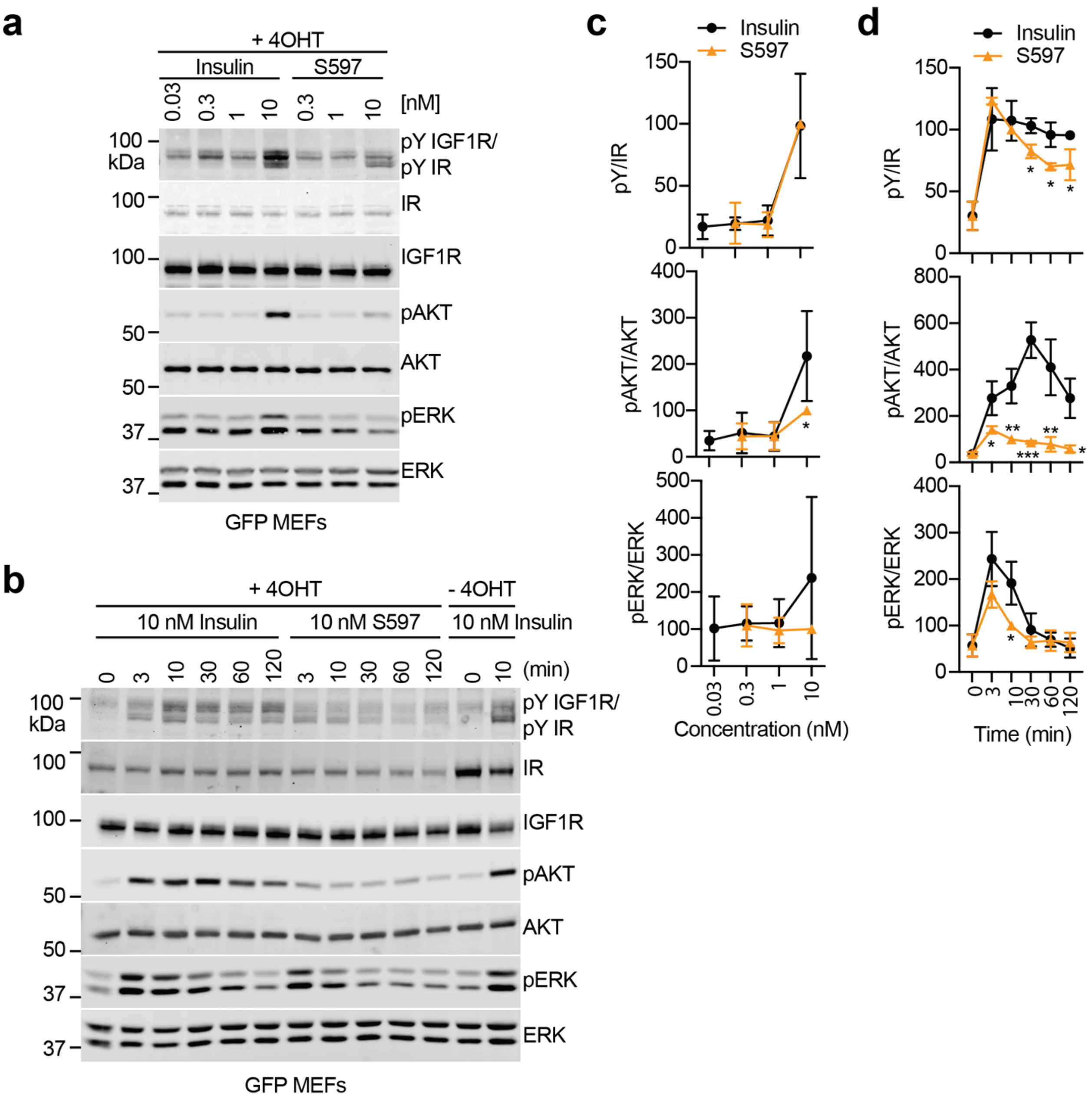

Supplementary Figure 8. The characterization of the tamoxifen-inducible IR knockout mouse embryonic fibroblasts expressing only GFP (GFP MEFs).

a. IR signaling in GFP MEFs treated with the indicated concentrations of insulin or S597 for 10 min. Cell lysates were blotted with the indicated antibodies. 4OHT, 4-Hydroxytamoxifen.

b. IR signaling in GFP MEFs treated with 10 nM insulin or S597 for the indicated times. 4OHT, 4-Hydroxytamoxifen.

c. Quantification of the western blot data shown in (**a**). Levels of protein phosphorylation were normalized to total protein levels and shown as intensities relative to that in 10 nM S597-treated cells. Mean ± SD. Each experiment was repeated five times. Significance calculated using two-

tailed Student's *t-test*; between insulin and S597 in the indicated concentrations, *p<0.05. The exact p values are provided in the source data.

d. Quantification of the western blot data shown in (**b**). Levels of protein phosphorylation were normalized to total protein levels and shown as intensities relative to that in 10 nM S597-treated cells for 10 min. Mean ± SD. Each experiment was repeated three times. Significance calculated using two-tailed Student's *t-test*; between insulin and S597 in the indicated time points, *p<0.05, **p<0.01, and ***p<0.001. The exact p values are provided in the source data. Source data are provided as a Source Data file.

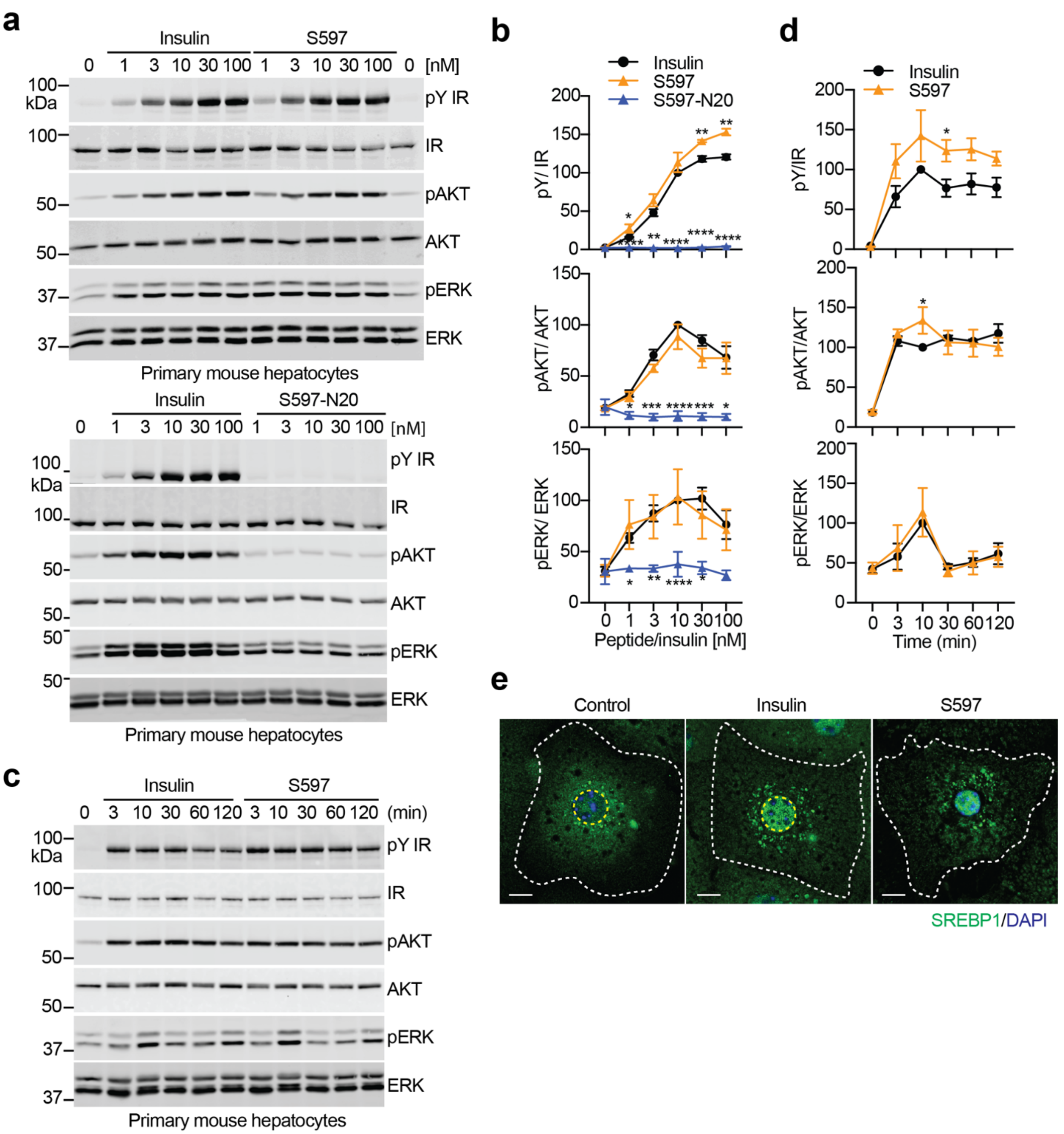

Supplementary Figure 9. The action of S597 in IR signaling and SREBP1 activation.

a. IR signaling in primary mouse hepatocytes treated with the indicated concentrations of insulin, S597 or S597-N20 for 10 min. Cell lysates were blotted with the indicated antibodies.

b. Quantification of the western blot data shown in (**a**). Levels of protein phosphorylation were normalized to total protein levels and shown as intensities relative to that in 10 nM insulin-treated cells. Mean ± SD. For insulin, n=5 independent experiments; S597, n=3; S597-N20, n=2.

Significance calculated using two-tailed Student's *t-test* with the insulin set as the control. $*p<0.05$; $**p<0.01$, $***p<0.001$, and $****p<0.0001$.

c. IR signaling in primary mouse hepatocytes treated with 10 nM insulin or S597 for the indicated times. Cell lysates were blotted with the indicated antibodies.

d. Quantification of the western blot data shown in (**c**). Levels of protein phosphorylation were normalized to total protein levels and shown as intensities relative to that in 10 nM insulin treated cells for 10 min. Mean ± SD. For pY/IR, insulin, n=4 independent experiments and S597, n=3; for pAKT/AKT, insulin, n=6 and S597, n=4; for pERK/ERK, insulin, n=4 and S597, n=3. Significance calculated using two-tailed Student's *t-test* with the insulin set as the control. $*p<0.05$.

e. Insulin- or S597-mediated nuclear translocation of SREBP1 in primary mouse hepatocytes. Cell and nuclear boundaries were noted as white dashed lines. Control (PBS), n=115; Insulin, n=221; S597, n=217. Scale bar, 10 μm. Source data are provided as a Source Data file.

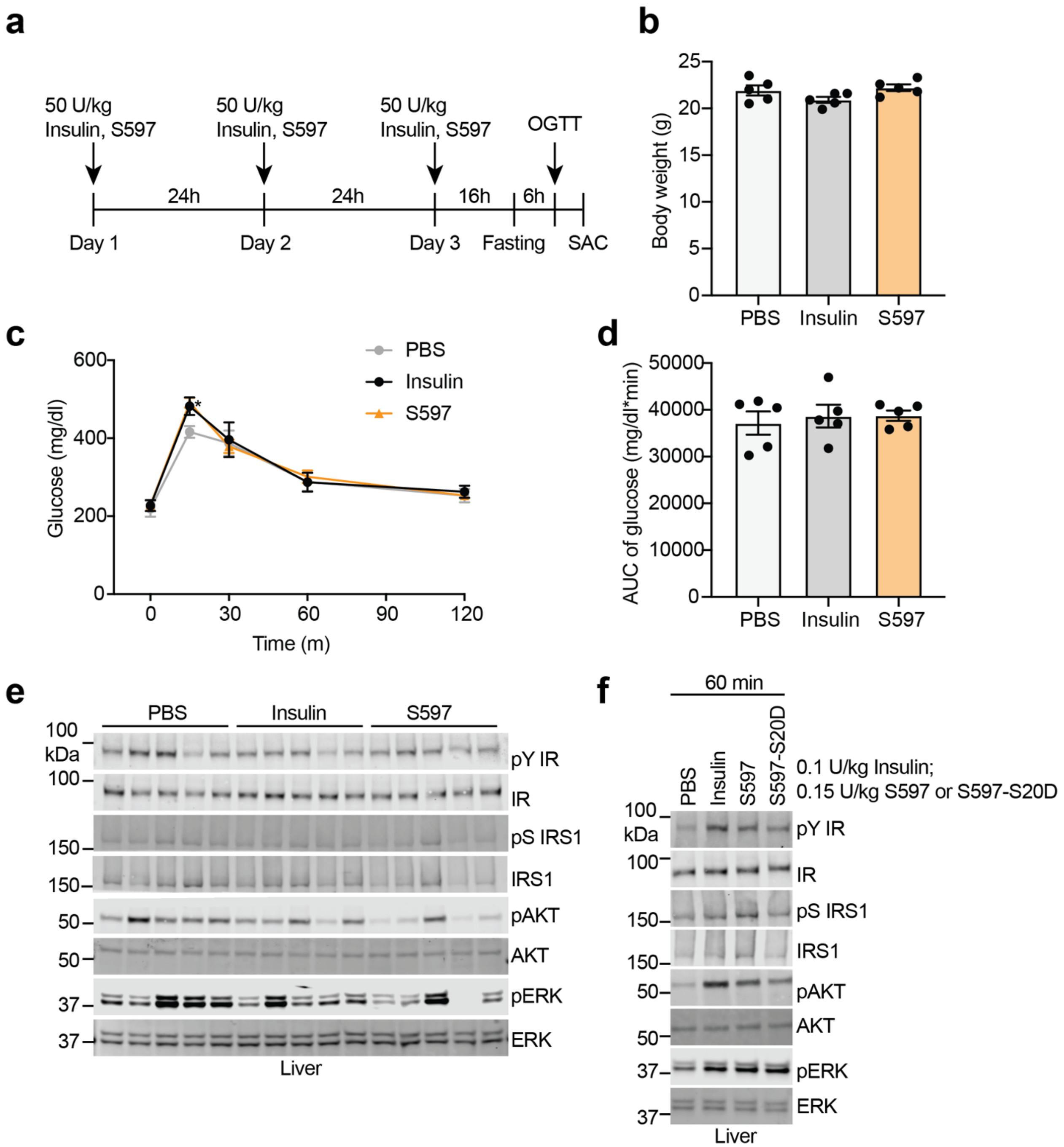

Supplementary Figure 10. The effects of chronic treatment with S597.

a. A schematic representation of the S597 treatment. Male C57BL/6J mice were treated with vehicle (PBS), insulin (50U/kg body weight), or S597 (50U/kg) once daily for three days. OGTT, oral glucose tolerance test; SAC, sacrifice. N=5 mice each.

b. Body weights measured before OGTT. Mean ± SEM. N=5 mice each. Significance calculated using two-tailed Student's *t-test.* No significant.

c. Oral glucose tolerance test (2g glucose/kg body weight) after 6 hr fast. Mean ± SEM. N=5 mice each. Significance calculated using two-way ANOVA followed by Tukey's multiple comparisons test. Between PBS and S597, *$p<0.05$. The exact p values are provided in the source data.

d. Area under curve (AUC) for blood glucose during OGTT (**c**). Mean ± SEM. N=5 mice each. Significance calculated using two-tailed Student's *t-test.* No significant.

e. Western blots of liver lysates at the completion of OGTT. Each lane contains lysate from an individual mouse. N=5 mice each.

f. Male C57BL/6J mice were fasted for 14 hr and then injected with vehicle (PBS), insulin (0.1U/kg), S597 (0.15U/kg), or S597-S20D (0.15U/kg). One hour later livers were collected. Western blots of liver lysates are shown. Each lane contains lysate from an individual mouse. N=1 mouse each. Source data are provided as a Source Data file.
